# Motor Cars for Doctors

**Published:** 1906-04-28

**Authors:** 


### MOTOR CARS FOR DOCTORS.

### HINTS ON BUYING A CAR.

One of the first hints that should be given is, Do not buy too cheaply, and, above all, do not buy a car with too low a power. Some of the members of the medical profession who purchased motor-cars a few years ago complained of having considerable trouble with them, and in some cases were obliged to abandon their use. In the great majority of cases the cause was, the car was of too low a power. Some of the earlier cars were heavier than improved appliances have enabled makers to produce since, and in addition they were provided with engines of much lower power than is now common. Six horse-power appears to be the minimum that should be available in a car such as a doctor could use, while 7 h.p. or 8 h.p. is better, and from that up to 16 h.p. is suitable for the majority of cars that doctors would use. With heavier cars, for special purposes, greater power should be employed. Higher power has the great advantage, if the engine is arranged on the latest modern lines, with control of the supply of petrol and air to the engine, that hills of moderate gradient may be climbed without the aid of the low-speed gear. The gearing of the petrol car, it should be understood, is its weak point. It swallows up a certain amount of power, often as much as 50 per cent.—33 per cent. is quite common —and the more the gear is used the greater is the chance of the power it absorbs increasing. The teeth of the wheels get chipped, and do not mesh properly after a time, and power is wasted. The importance of this will be understood when it is mentioned that with a 50 per cent. loss in transmission the power actually delivered at the wheels of the car is only half that developed in the engine; or, to put it in another way, if there was no loss, the engine might be half the size. It is the power that is actually delivered at the driving wheels of the car that is important, and that is a point in which the steam car has the advantage over the petrol car. There is no gearing in the steam car except the differential. The real test of power is the proportion it bears to the weight of the car, engine, and load. Hill-climbing is the thing motor-cars have most to fear, as it absorbs the most power. The car has to be lifted vertically the perpendicular height of the hill, and in addition the ordinary friction of the road has to be overcome, bearing in mind that a certain amount of friction is necessary, or the car cannot climb at all. And hill-climbing is solely a question of power per cwt., including the weight of the engine, the car itself, and its load. The power per cwt. of the cars on the market runs from 1 h.p. per 130 lb. down to 1 h.p. per 170 lb.

The matter of cheapness is one that is very difficult to deal with. There is always a certain price that a firm must receive, on the average, for its goods, or it must cease to trade, and it is greatly to the disadvantage of the owner of a motor-car for the maker of the car to go out of business. In motor-cars, as in so many things into which complicated machinery enters, the repairs are the most important in the whole affair. It is not only that the bill for repairs should be small that is important, but it is of far more importance that repairs shall be obtainable, and that spare parts in particular shall be obtainable. It is usually a good guide to obtain prices from two or three makers for the same thing. They will differ, but not by much; and if the price of one firm is very much below that of the others, the matter should be inquired into. It does not follow that the low-priced car is badly made, or has low power per cwt., or is weakly constructed. There are always firms who are moving ahead of their competitors, and who by improved appliances, improved design, and in other ways are able to reduce the cost; but the matter should be as carefully gone into as the opportunities at the disposal of the buyer will allow. There are two methods of going into the matter—taking the advice of an expert, and taking the experience of someone who has used a similar car. The expert is quite satisfactory, provided he is really what he claims to be. Many men claim to be experts upon every branch of engineering who have really no claim to the title; they can only be safe guides in one branch. An expert in motor-cars should be a trained engineer who has specialised in motor-car work, and who is thoroughly conversant with the manufacture and running of every car on the market. A man engaged in the manufacture of cars, if he will give a really unbiassed opinion, is perhaps the best expert obtainable. The doctor should be careful not to try to economise by employing a workman who says he knows all about it. The expert, if he really knows his work, is well worth his fee. Either have a good expert or leave it alone. The other plan, the experience of a friend, is fairly safe, but requires some caution. There are two classes of men, from a mechanician's point of view—those who have a turn for machinery and those who have not. Wives express it by saying that Tom will do anything about the house, while Jack will not even knock a nail in. Tom's advice about motor-cars, if he has owned one himself, will be very valuable; but it will not be safe to follow him in buying a car. Almost any car would come out right in his hands. He will be only too delighted at having something to puzzle over and put right. If Jack has had a car, and it has gone well, it is perfectly safe to buy a similar one. Another point of importance is, manufacturers vary in their statements of horse-power. Some makers are very liberal in the matter on the side of the buyer; others are very liberal on their own side. Obviously when a buyer is estimating the horse-power per cwt., the firm who have taken the latter course will apparently be giving most for the money. A fairly safe guide is the sizes of the engines. Horse-power in any engine is measured by the area of the cylinder in square inches, multiplied by the length of stroke, by the number of revolutions per minute, and by the mean effective pressure in the cylinder during the stroke. For the purpose of comparison, in petrol cars the last factor may be omitted, and the figure representing the product of the first three compared in any two or more cars represented to have the same horse-power. The product of the length of stroke and the number of revolutions per minute will be found to be about the same in all cases. It measures the speed at which the pistons of the engines are travelling, and is governed by the question of efficient lubrication. Some firms run their engines at apparently a higher speed than others, a greater number of revolutions per minute, but it will be found that the stroke of the engine is then shorter. Where a firm can, by an improved method of lubrication, safely run at a higher piston speed, they can produce a lighter engine and a cheaper one. Where there are two or more cylinders, the power is approximately as many times that of a single cylinder. In comparing the power by the sizes of the cylinders, steam cars must not be compared with petrol. Petrol engines only receive an impulse every four strokes, while steam engines receive one at every stroke, and in addition the smaller number of gear wheels required with the steam engine gives a higher proportion of the power of the engine at the driving wheels of the car. The question of one, two, or more cylinders is also an important one. Two cylinders are always better than one, because in all cases they give what engineers call a more even turning moment, the work done by the engines is distributed over different parts of the revolution of the crank shaft, as there are more and more cylinders, and the engine itself becomes more flexible, the speed of the car more easily controlled without the aid of the change-speed gear. The majority of cars fitted for doctors' use are constructed with either one or two cylinders. The money paid for the second cylinder is well laid out.

### Steam *v.* Petrol or Electric Cars.

The electric car is the most comfortable and the most convenient where arrangements can be made for charging the accumulators conveniently, and where the small radius of operation is not a drawback. As between the other two forms, petrol appears to have taken the lead, mainly, we believe, because the motoring public have been frightened by the failures of some of the boilers that were put on the market a few years since. Some steam cars have been uniformly successful, and the steam car has a great deal to recommend it. It is less complicated, its power is more efficiently utilised, its running is more even, its speed more easily controlled, and it starts and stops more easily and quickly.

### Tyres.

There is only one opinion on this matter. The pneumatic tyre is the one and only tyre for the doctor's car. At present it is expensive, both in first cost and in upkeep, but the latter can be very much reduced by careful driving. Meanwhile the pneumatic car renders riding easier to the human who is carried, and to the engine and other machinery that carries him. With solid tyres, every inequality of the road, every stone, etc., is painfully apparent. With the pneumatic tyre they are very largely masked. The tyre swallows up small inequalities by its own resilience. The tyre is practically a bag of air, which is compressed when the tyre passes over any inequality, the compressed air in the bag serving to gently bring the tyre back to its normal condition when the inequality is passed. The use of pneumatic tyres enables makers of motor-cars to lighten them very considerably, and it prolongs the life of the machinery by relieving it from shocks.

Other points that are of importance in choosing a car are: There should be facilities for inspecting all the working parts of the machinery, and the arrangements for lubrication should be such that the lubricant is easily placed in position, and that it is easily seen if it is finding its way between all the rubbing surfaces. The wheel bas of the car should be neither too long nor too short. If it is too long, corners are awkward to negotiate, if too short it tends to side slip. By wheel base is meant the distance between the axles of the front and rear wheels. Wind screens and hoods take from the speed of the car by the resistance they offer to the wind. The bodies of broughams and landaulettes also add to the weight of a car, and to the engine power required for a given speed.

### The Guarantee.

The guarantee given by makers is of importance. This question has come up with every new machine that has been placed on the market. The safest and cheapest guarantee for buyer and seller is an absolute one for a certain time, six or twelve months, wilful damage or extraordinary accidents excepted. Guarantees are among the most fruitful sources of disputes. A good firm who liberally interprets the above guarantee saves considerably in the costs of disputes, and obtains more business than its competitors who refuse to give a similar one. In the early days of many new machines disputes were very rife, till the twelve months' guarantee was adopted, and after that there was very little trouble.

### The Change-speed Gearing.

One of the usual engineering battles is raging around this question. The great majority of petrol cars are fitted with spur gearing, enclosed in the box described, and with four possible speeds, but a few cars are fitted with epicyclic, which its backers claim is more efficient than spur. The Durea company, who are its principal advocates, give the results of tests by an expert, showing an advantage in favour of epicyclic, as 85 per cent. to 69 per cent., which means that of, say, 10 h.p., at the crank shaft, 8.5 h.p. would be delivered to the wheels with epicyclic, where only 6.9 h.p. would be delivered with spur gearing, the latter tending to become less efficient, as explained, by the chipping of the teeth. On the other hand, the supporters of spur gearing claim that the rival method involves high speed of some of the axles, and consequent heating. The real reason, we believe, for the adoption of the different forms is, it is easier to fit epicyclic to horizontal engines than spur gearing, while it is easier to fit spur gearing to vertical engines. We believe also that epicyclic gear is not so well understood by mechanical engineers as the other.

